# The publics' understanding of daily caloric recommendations and their perceptions of calorie posting in chain restaurants

**DOI:** 10.1186/1471-2458-10-121

**Published:** 2010-03-09

**Authors:** Sara N Bleich, Keshia M Pollack

**Affiliations:** 1 Department of Health Policy and Management, Johns Hopkins Bloomberg School of Public Health, Baltimore MD, USA; 2 Center for Health Disparities Solutions, Johns Hopkins Bloomberg School of Public Health, Baltimore MD, USA; 3 Center for Injury Research and Policy, Johns Hopkins Bloomberg School of Public Health, Baltimore MD, USA

## Abstract

**Background:**

Calorie posting in chain restaurants has received increasing attention as a policy lever to reduce energy intake. Little research has assessed consumer understanding of overall daily energy requirements or perceived effectiveness of calorie posting.

**Methods:**

A phone survey was conducted from May 1 through 17, 2009 with 663 randomly selected, nationally-representative adults aged 18 and older, including an oversample of Blacks and Hispanics in the United States. To examine differences in responses by race and ethnicity (White, Black, and Hispanic) and gender, we compared responses by conducting chi-squared tests for differences in proportions.

**Results:**

We found that most Americans were knowledgeable about energy requirements for moderately active men (78%) and women (69%), but underestimated energy requirements for inactive adults (60%). Whites had significantly higher caloric literacy and confidence about their caloric knowledge than Blacks and Hispanics (p < 0.05). As compared to their counterparts, Blacks, Hispanics and women reported a significantly higher likelihood of eating at a chain restaurant and of selecting lower calorie foods where caloric information was posted. Most Americans favored the government requiring chain restaurants to post calorie information on menus at the point of purchase (68%). Support for government mandated calorie posting in chain restaurants was significantly higher among Blacks, Hispanics and women as compared to their counterparts. The public was divided about the mode of caloric information that would best help them make a lower calorie decision; a third favored number of calories (35%) which is the current standard mode of presenting caloric information in chain restaurants, a third favored a physical activity equivalent (26%), and a third favored percentage of total energy intake (39%).

**Conclusion:**

Mandating calorie posting in chain restaurants may be a useful policy tool for promoting energy balance, particularly among Blacks, Hispanics and women who have higher obesity risk.

## Background

As experts increasingly point to the environment as the primarily driver of obesity [1-3], calorie posting on menus or menu boards at restaurants (hereafter referred to as "calorie posting"), alongside price, has received growing attention as a potential policy lever to reduce energy intake and promote energy balance (equilibrium between energy intake and energy expenditure). To date, calorie posting legislation has passed in two cities (New York City, Seattle) and one state (California) and is currently under consideration in more than 20 states, cities and counties in the United States [4]. In the United Kingdom, the Food Standards Agency has asked fast food restaurants to voluntarily post calories on menus [5]. Theoretically, calorie posting should have a larger impact on individuals with greater caloric literacy (defined as an understanding of federal energy intake guidelines) given that perceived risk is a critical precursor to behavior change [6].

Shifts in consumption patterns and poor nutritional literacy give strong justification for calorie posting, particularly in chain restaurants which account for approximately half of all restaurant visits [7]. Over the past three decades, Americans have greatly increased the percent of their food dollars spent on food away from home; from roughly a third in 1970 to a half in 2001 [8]. Foods eaten away from home, especially fast food, are more energy dense and have a higher percentage of fat and sugar [9]. In addition, foods with a higher energy density are associated with excess energy consumption [10].

A key challenge to limiting energy intake is the publics' significant underestimation of the amount of calories in the foods they consume [11,12]. A recent study that asked participants to estimate the caloric content of nine restaurant entrées found that 90% underestimated the calorie content of less-healthy items by an average of more than 600 kcal [12]. In the same study, when calorie information was provided on food items, consumers chose high calorie items roughly a third less often. Since most surveys evaluating nutrition literacy primarily focus on nutrient composition (e.g., percent fat, percent fiber) rather than overall energy needs (e.g., "caloric count"), they are of limited usefulness for informing current efforts around calorie posting.

To our knowledge, little research has assessed consumer understanding of overall daily energy requirements or perceived effectiveness of calorie posting [13]. This study - which is the first to use nationally-representative public opinion data to address this research question - will fill this gap in the knowledge base. We hypothesized that the publics' caloric literacy (e.g., understanding of federal daily caloric recommendations) will be poor, and that Americans will strongly support calorie posting initiatives. In addition to looking at overall knowledge patterns and views about calorie posting, we additionally stratified our results by race/ethnicity and gender given the disproportionate impact of obesity on minorities and women [14]. Findings from this study may be useful for policy makers looking to pass calorie posting legislation and for researchers interested in exploring the impact of such initiatives.

## Methods

### Data

The survey was designed by the Johns Hopkins Bloomberg School of Public Health and conducted from May 1, 2009 through May 17, 2009. The study sample included 663 adults aged 18 and older in the United States, including an oversample of Blacks and Hispanics. All together, 119 Blacks and 103 Hispanics were interviewed. We performed a power analysis under the assumption that we would have 100 Blacks, 100 Hispanic and 250 White participants and an effect size of 0.12. Survey respondents were selected from households across the country using a fully replicated, stratified, single stage, random-digit-dialing sampling strategy. Within each sample household, a single respondent was randomly selected. The data were weighted to account for the disproportionate probability of household selection attributable to multiple telephone lines and the probability associated with the random selection of an individual household member. In addition, the data were weighted by age, sex, race/ethnicity, education, and census division to ensure nationally representative findings. The sample error for 683 respondents was ± 4 percentage points. Interviews were administered by International Communications Research (ICR) and lasted approximately 7 to 10 minutes in duration. Given that we do not have a response rate for this survey, the accuracy of the data is determined by the representativeness of the sample. This was ensured by re-weighting, and analyses have shown that the results of statistical re-weighting of the data are similar to those of an analysis based on the higher response rate in opinion surveys of long duration [15-19].

This study was approved by the Johns Hopkins Bloomberg School of Public Health Institutional Review Board.

### Survey Questions

To determine the accuracy of consumer knowledge of daily energy requirements, we used the current U.S. federal dietary guidelines which are calculated based on an individual's gender, age and physical activity level [20]. For simplicity, we aggregated the recommendations and asked respondents their perceptions of caloric recommendations for moderately active men, moderately active women and inactive adults. Respondents were asked, "Now, thinking about [moderately active men/moderately women/inactive adults], what is the recommended daily number of calories - less than 1500 calories, 1500 to less than 3000 calories, 3000 to less than 4500 calories, or 4500 calories or more?" For each question, we defined the level of activity (e.g., moderately active or inactive) at the end of the question (e.g., "Moderate physical activity included exercises such as brisk walking, bicycling or gardening for about 30 minutes each day.") We created the response categories such that the correct answer for each question about recommended energy requirements was the same (e.g., 1500 to less than 3000 calories). The caloric knowledge questions were developed with consultation from the Harvard Opinion Research Program.

In the survey instrument, we included both open-ended and close-ended questions regarding caloric knowledge. We purposely asked the open-ended question first so as not to prime the respondent with the response categories for the closed-ended questions. The results reported in this manuscript include only the responses to the close-ended caloric knowledge questions. For those questions, there we no missing values and the percentage of respondents who answered ''don't know'' or ''refused'' was less than ten percent for each question. So, we treat those responses as missing at random as they would have minimal contribution to any selection bias.

We additionally asked a series of questions to assess perceptions of caloric labeling in chain restaurants on menu boards alongside price. The questions specifically asked respondent's their beliefs about perceived usefulness of calorie posting, personal knowledge of energy requirements, how calorie posting would impact their behavior in a chain restaurant, preferred type of calorie posting, calorie posting legislation and government responsibility for calorie posting (see Appendix for exact wording of survey questions). In each question which mentioned chain restaurants, we gave respondents' the examples of McDonalds and Subway. To assess respondents' preferred type of calorie posting, they were asked about three different possibilities: 1) number of calories (which is the dominant way chain restaurants display caloric information); 2) number of calories translated into a physical activity equivalent such as eating a hamburger would require 80 minutes of brisk walking to burn off the calories and 3) number of calories translated into a percentage of total daily intake such as eating a hamburger is equal to 10 percent of total daily calories.

Self-reported height and body weight were used to calculate body mass index (BMI) categories. Healthy weight was defined as BMI from 18.5 to 24.9, overweight was defined as BMI from 25 to 29.9, and obese was defined as BMI ≥ 30 [21].

### Statistical Analysis

To examine differences by race and ethnicity (White, Black, and Hispanic) and gender, we compared responses by conducting chi-squared tests for differences in proportions. We considered p < 0.05 to be statistically significant. For all analyses, we accounted for the study design and weighted the data to be representative of the general population. Analyses were performed using the STATA, version 9.2 software package (StataCorp LP, College Station, TX).

## Results

### Characteristics of the study sample

Table 1 reports the characteristics of the study sample. Approximately half of respondents (49%) were female, under 45 years of age (46%), married or living with a partner (57%), employed full-time (45%), or had more than a high school degree (55%). Roughly two-thirds of the study sample was White (68%). One third of the sample (33%) reported being overweight, and one quarter (24%) reported being obese. The prevalence of overweight and obesity was similar to national estimates [22]. Two-thirds of the study sample reported visiting fast food restaurants at least once weekly (70%).

**Table 1:** Characteristics of the study sample

	%	*N*	Missing (%)
Gender			
Female	49	347	0.0
Male	51	316	
Race			
White	68	414	7.6
Black	11	119	
Hispanic	13	103	
Age, y			
18-44	46	193	0.0
45-64	38	278	
65+	16	192	
Marital status			
Married/living with partner	60	400	0.3
Single, never married	19	91	
Separated/divorced	13	97	
Widowed	8	73	
Employment status			
Employed full-time	45	283	0.0
Employed part-time	14	81	
Retired	17	180	
Other (e.g., homemaker, student)	24	119	
Household income			
Less than $30,000	34	206	0.0
$30,000 to less than $75,000	32	214	
More than $75,000	33	243	
Education			
Less than high school	14	49	0.0
High school diploma	31	205	
More than high school	55	408	
BMI category			
Healthy weight	38	241	5.6
Overweight	33	236	
Obese	24	153	
Weekly visits to fast food restaurants			
None	29	234	0.5
1-2	51	314	
3-4	13	74	
5+	6	36	

*Notes*: Numbers may not add up to 100% because of rounding. Data are weighted to be representative of the national population. The BMI categories are defined as follows: healthy weight (BMI, 18.5 to 24.9 kg/m2), overweight (BMI, 25.0 to 29.9 kg/m2), and obese (BMI, ≥ 30 kg/m2). Sample sizes vary across categories because of missing data.

### American reports of daily recommended energy requirements

Public perceptions of daily energy requirements are presented in Table 2. For each category (moderately active men, moderately active women, inactive adults), the response category consistent with current federal dietary guidelines is 1500 to less than 3000 calories [20]. Most Americans correctly identified the recommended energy requirements for moderately active men (78%) and moderately active women (69%), but only a third correctly identified the energy requirements for inactive adults (35%). Reports of daily recommended energy requirements for moderately active men, moderately active women and inactive adults differed significantly by race/ethnicity and age (p < 0.05). Reports of daily recommended energy requirements for inactive adults differed significantly by gender. We observed no differences in caloric knowledge by BMI category or educational attainment.

**Table 2:** American reports of daily recommended energy requirements

	Knowledge of energy requirements								
	
	Moderately active men			Moderately active women			Inactive adults		
	
	< 1500 kcal	1500 to < 3000 kcal	> 3000 kcal	< 1500 kcal	1500 to < 3000 kcal	> 3000 kcal	< 1500 kcal	1500 to < 3000 kcal	> 3000 kcal
Total	8	78	14	26	69	5	**60**	**35**	**5**

Gender									
Female	6	79	15	31	65	4	**73**	**25**	**2**
Male	10	76	14	20	74	5	**48**	**44**	**8**
*P-value*	0.276			0.082			**<0.001**		

Race	**7**	**83**	**10**	**23**	**74**	**3**	**64**	**34**	**2**
White	**7**	**83**	**10**	**23**	**74**	**3**	**64**	**34**	**2**
Black	**14**	**65**	**21**	**43**	**49**	**7**	**59**	**28**	**13**
Hispanic	**14**	**60**	**26**	**30**	**64**	**6**	**55**	**35**	**10**
*P-value*	**0.001**			**0.012**			**<0.001**		

Age, y									
18-44	**5**	**74**	**21**	**19**	**73**	**8**	**52**	**41**	**7**
45-64	**9**	**81**	**9**	**28**	**70**	**2**	**65**	**31**	**4**
65+	**13**	**79**	**8**	**39**	**58**	**3**	**73**	**26**	**2**
*P-value*	**<0.001**			**<0.001**			**0.010**		

Education	8	74	18	36	57	7	52	44	4
Less than high school	8	74	18	36	57	7	52	44	4
High school diploma	12	69	19	31	66	3	63	29	8
More than high school	6	83	11	20	74	5	60	36	4
*P-value*	0.084			0.156			0.379		

BMI category									
Healthy weight	7	82	11	25	70	5	59	34	7
Overweight	11	75	14	25	70	5	59	36	5
Obese	7	74	19	27	69	4	60	38	3
*P-value*	0.295			0.984			0.586		

*Notes*: Numbers may not add up to 100% because of rounding. Data are weighted to be representative of the national population. P-values for chi-squared tests were calculated among demographic and bodyweight categories. Sample sizes vary across categories because of missing data.

### The public's self-perceived caloric knowledge and perceived usefulness of calorie posting in chain restaurants

Table 3 presents public views on self-perceived caloric knowledge and perceived usefulness of calorie posting in chain restaurants. Half of Americans reported knowing enough about daily energy requirements to make lower calorie choices (56%), most reported that being told the calorie count of foods at the point of purchase in a chain restaurant would be very or somewhat useful (76%), and about half reported that they would be more likely to eat at a chain restaurant which reported caloric information on the menus alongside the price of each food item (51%). Whites, older adults and individuals with more than a high school education reported being more confident about their caloric knowledge than their counterparts. Women and middle age individuals reported being more likely than their counterparts to perceive calorie posting as useful. Women, Blacks and Hispanics reported a higher likelihood than their counterparts of eating at a chain restaurant with caloric information on the menu.

**Table 3:** The public's self-perceived caloric knowledge and views on usefulness of calorie posting in chain restaurants

	Know enough about energy requirements to make lower calorie choices		Usefulness of calorie count to food choice at the point of purchase in chain restaurants				Likelihood of eating at chain restaurants with calorie information on the menu		
	
	Yes	No	Very useful	Somewhat useful	Not very useful	Not at all useful	More likely	Less likely	Neither
Total	**56**	**44**	**44**	**32**	**6**	**18**	**51**	**20**	**29**

Gender									
Female	**62**	**38**	**52**	**32**	**6**	**11**	**57**	**19**	**23**
Male	**51**	**49**	**36**	**32**	**6**	**26**	**45**	**21**	**34**
*P-value*	**0.026**		**0.001**				**0.028**		

Race	**63**	**37**	42	35	6	17	**46**	**20**	**33**
White	**63**	**37**	42	35	6	17	**46**	**20**	**33**
Black	**38**	**62**	49	26	4	21	**56**	**29**	**15**
Hispanic	**40**	**60**	53	21	5	21	**61**	**17**	**22**
*P-value*	**< 0.001**		0.200				**0.010**		

Age, y	**49**	**51**	**38**	**37**	**4**	**20**	52	22	26
18-44	**49**	**51**	**38**	**37**	**4**	**20**	52	22	26
45-64	**63**	**37**	**50**	**29**	**6**	**15**	52	19	30
65+	**61**	**39**	**46**	**24**	**10**	**20**	46	19	35
*P-value*	**0.010**		**0.031**				0.502		

Education	**39**	**61**	47	25	2	26	48	27	25
Less than high school	**39**	**61**	47	25	2	26	48	27	25
High school diploma	**44**	**56**	39	36	7	19	53	19	29
More than high school	**68**	**32**	46	31	6	17	51	19	30
*P-value*	**< 0.001**		0.421				0.781		

BMI category	58	42	43	30	6	21	58	19	23
Healthy weight	58	42	43	30	6	21	58	19	23
Overweight	53	47	40	37	6	17	42	25	33
Obese	57	43	51	27	4	18	52	17	32
*P-value*	0.704		0.559				0.060		

*Notes*: Numbers may not add up to 100% because of rounding. Data are weighted to be representative of the national population. P-values for chi-squared tests were calculated among demographic and bodyweight categories. Sample sizes vary across categories because of missing data.

### American perceptions of calorie posting in chain restaurants

Table 4 presents American views on calorie posting in chain restaurants. Roughly two-thirds of Americans reported that calorie posting would encourage them to select a lower caloric food (60%) while a third reported that it would not influence their purchase at all (38%). Less than half reported they would feel guilty if calories were posted on the menu and they picked a higher calorie food (40%). When asked whether they would pay more attention to price or calories in a chain restaurant with calories posted on the menu alongside the price, Americans reported being pretty evenly divided between paying attention to price (44%) and paying attention to calories (47%).

**Table 4:** American perceptions of posting caloric information in chain restaurants

	Calorie posting in the menu board next to the price would encourage you to...			Feel guilty for picking a higher calorie food if calories were posted		In a chain restaurant with calories posted on the menu alongside price, more likely to pay attention to...		
	
	Select a higher calorie food	Select a lower calorie food	Not influence purchase	Yes	No	Price	Calories	Both Equally
Total	**2**	**60**	**38**	**40**	**60**	**44**	**47**	**9**

Gender								
Female	**0**	**72**	**27**	**49**	**51**	**35**	**58**	**7**
Male	**4**	**48**	**48**	**32**	**68**	**53**	**38**	**10**
*P-value*	**< 0.001**			**0.001**		**< 0.001**		

Race								
White	**1**	**59**	**40**	38	62	45	46	9
Black	**5**	**62**	**33**	40	60	36	53	10
Hispanic	**1**	**63**	**36**	43	57	47	51	3
*P-value*	**0.027**			0.735		0.250		

Age, y								
18-44	**4**	**54**	**42**	43	57	48	45	6
45-64	**0**	**65**	**35**	37	63	41	49	10
65+	**1**	**63**	**35**	39	61	39	49	12
*P-value*	**0.026**			0.335		0.359		

Education								
Less than high school	**4**	**27**	**69**	**25**	**75**	60	36	5
High school diploma	**1**	**66**	**33**	**45**	**55**	49	44	8
More than high school	**3**	**65**	**33**	**42**	**58**	38	52	10
*P-value*	**< 0.001**			**0.044**		0.089		

BMI category								
Healthy weight	3	57	40	38	62	43	48	9
Overweight	3	59	38	43	57	43	51	6
Obese	1	60	40	38	62	47	42	11
	0.832			0.630		0.573		

*Notes*: Numbers may not add up to 100% because of rounding. Data are weighted to be representative of the national population. P-values for chi-squared tests were calculated among demographic and bodyweight categories. Sample sizes vary across categories because of missing data.

American perceptions of calorie posting differed significantly by sociodemographic characteristics. Women (as compared to men), Black and Hispanics (as compared to Whites), older adults (as compared to adults aged 45 and below), and more educated adults (as compared to those with less than a high school education) were significantly more likely to report that calorie posting would encourage them to select a lower calorie food. Women and more educated individuals, as compared to their counterparts, were significantly more likely to report feeling guilty for selecting a higher calorie food if calories were posted on the menu. Women were also significantly more likely than men to report paying attention to calories if they were posted on the menu alongside price.

### Public preferences for the type of calorie posting and views on calorie posting policies

The public was evenly divided about the mode of caloric information that would best help them make a lower calorie decision in a chain restaurant; a third favored number of calories (35%) which is the current standard mode of presenting caloric information in chain restaurants, a third favored a physical activity equivalent (26%), and a third favored percentage of total daily energy intake (39%). Most Americans favored the government requiring chain restaurants to post calorie information on menus at the point of purchase (68%). Support for government mandated calorie posting in chain restaurants was significantly higher among Blacks, Hispanics and women as compared to their counterparts. A majority of Americans believed that either the federal (29%) or the state/local government (48%) should bear the responsibility for requiring chain restaurants to post caloric information.

## Discussion

The central aims of this study were to assess the public's knowledge of daily energy requirements and their beliefs about the perceived usefulness of caloric information on chain restaurant menu boards - overall and among subgroups at higher obesity risk. This is the first national poll to assess public beliefs about calorie posting initiatives which require some chain restaurants to post caloric information on menus or menu boards alongside price.

Overall, we found that most Americans were knowledgeable about energy requirements for moderately active men and women, but tended to underestimate energy requirements for inactive adults. Only about half of the public perceived themselves as sufficiently knowledgeable to make lower calorie choices in chain restaurants. Americans expressed a positive attitude towards calorie posting in chain restaurants; a majority viewed it as very or somewhat useful, reported being more likely to eat in a chain restaurant with calories posted in the menu and reported being more likely to select a low calorie food where calories were posted. Given that price, convenience and taste [23,24] are the major motivators for food purchasing decisions, our finding that Americans were evenly divided between purchasing an item based on calories or price is very encouraging for calorie posting initiatives.

Our findings also suggest that calorie posting initiatives may be more salient among certain sociodemographic groups. Blacks, Hispanics and women, who are disproportionately impacted by obesity [14], were more likely than their counterparts to perceive calorie posting as very useful, to report they would eat in a chain restaurant with calorie posting and to report that caloric posting would encourage their selection of a lower calorie food. Therefore, mandatory calorie posting initiatives may be more salient among women and racial/ethnic groups.

These results further suggest that the best mode for communicating caloric information in chain restaurants may not be calorie count (e.g., a hamburger is equal to 300 kcal) - the dominant mode of communicating caloric information in chain restaurants to date. Rather the public is pretty evenly divided between three possible presentation formats: number of calories, physical activity equivalent, or percentage of total daily energy intake. Our finding that most Americans favor a mode of communication other than a calorie count is consistent with prior research suggesting that interpretational aids help consumers with nutritional labels [25]. It also may partially explain mixed findings on the impact of calorie labeling on purchasing behavior in New York City; some studies show an impact [26] whole others show none [27].

This study makes three important contributions to the evidence base. To our knowledge, this is the first study to assess consumer understanding of overall daily energy requirements and perceived effectiveness of calorie posting in a nationally representative study sample. Previous research has focused on sub-populations and small geographic areas [13]. Second, it identifies groups at higher risk for obesity who are most likely to be positively affected by calorie posting initiatives. Third, the results from this study may inform the recent legislative interest in mandating calorie posting in chain restaurants in the United States and other developed countries.

There are limitations to this analysis which deserve discussion. First, this cross sectional analysis only allows us to address associations. Second, we asked our respondents to think abstractly about how they might use caloric labeling at the point of purchase. Given that individuals are inclined to have an optimism bias - tendency to be overly positive about the outcome of their planned actions [28] - our results may be somewhat inflated. Third, that the public appeared to have a good understanding of the recommended federal energy requirements for moderately active adults but did not perceive themselves as sufficiently knowledgeable to make lower calorie choice, may be more related to the plethora of dietary guidelines based on the 2,000 kcal diet and less a reflection of true caloric knowledge. Fourth, because the range of calories in the correct response category for the caloric knowledge question was broad, our finding of relatively high caloric knowledge may be biased upwards. Fifth, given that the correct answer to the caloric literacy questions was the same for all groups (e.g., moderately active men, moderately active women and inactive adults), some respondents may have assumed the answer should change across groups. This would bias our results downwards and may partially explain our finding of low caloric literacy about inactive adults. Sixth, we relied on self-reported height and body weight to calculate body mass index which may lead to an underestimation of the obese population [29]. However, research suggests that the self-reported height and body weight bias do not differ by race/ethnicity[30] Seventh, random digit dialing, which was used to capture the study population, omits individuals who rely sole on cell phones or individuals who cannot afford a land line in their home. Finally, that McDonalds and Subway were provided as examples of chain restaurants in the survey questions may have affected respondent views on calorie posting. The direction of the bias would have depended on their perceptions of those chain restaurants.

Despite these limitations, this study has several strengths. It is consistent with state and local efforts in the United States to mandate calorie posting in chain restaurants as well as with the current federal priorities to reduce the prevalence adult obesity [31-33]. Second, it is very timely. Given wide-spread recognition among experts that the obesity epidemic is largely driven by environmental changes [1-3], there is considerable interest in designing effective societal-level interventions which reduce energy. Third, a better understanding of public perceptions about calorie posting may encourage policy makers to adopt this policy tool which is likely low cost, particularly for large food outlets with standard menus. In turn, the likely "calorie shock" about the high number of calories in food options, which has been reported in the mass media, may encourage restaurant chains to highlight lower calorie options and/or introduce healthier options. Some restaurants may also begin voluntarily providing caloric information. For example, YUM! Brands (owners of Pizza Hut and KFC) have announced plans to begin providing caloric information in their 20,000 outlets nationwide by 2011, regardless of legislative requirements.

The results from this study suggest the need for more research in two key areas. First, that a majority of Americans miscalculate the energy requirements of inactive adults (which characterizes most Americans [34]) suggests a poor understanding of energy balance. Second, more research is needed to understand whether the most effective mode for presenting consumers with calorie information and whether it varies by sociodemographic characteristics.

## Conclusion

To conclude, mandating calorie posting in chain restaurants may be a useful policy tool for promoting energy balance, particularly among Blacks, Hispanics and women who are at higher risk for obesity [14]. Improved knowledge about how to maximize the effectiveness of calorie posting in chain restaurants may encourage policy makers to adopt this relatively low cost policy tool.

## Competing interests

The authors declare that they have no competing interests.

## Authors' contributions

SNB and KMP conceived the study and developed the hypotheses. SNB analyzed the data. All authors contributed to the interpretation of study findings. SNB drafted the manuscript and all authors contributed to the final draft. All authors read and approved the final manuscript. SNB is the guarantor.

## Appendix: Question Wording

*Caloric literacy*

1. Now, thinking about **moderately **active men, what is the recommended daily number of calories - less than 1500 calories, 1500 to less than 3000 calories, 3000 to less than 4500 calories, or 4500 calories or more? Moderate physical activity includes exercises such as brisk walking, bicycling or gardening for about 30 minutes each day.

1 Less than 1500 calories

2 1500 to less than 3000 calories

3 3000 to less than 4500 calories

4 4500 calories or more

2. Now, thinking about **moderately **active women, what is the recommended daily number of calories - less than 1500 calories, 1500 to less than 3000 calories, 3000 to less than 4500 calories, or 4500 calories or more? Moderate physical activity includes exercises such as brisk walking, bicycling or gardening for about 30 minutes each day.

1 Less than 1500 calories

2 1500 to less than 3000 calories

3 3000 to less than 4500 calories

4 4500 calories or more

3. Now, thinking about **inactive **adults, what is the recommended daily number of calories - less than 1500 calories, 1500 to less than 3000 calories, 3000 to less than 4500 calories, or 4500 calories or more? Inactivity is defined as sitting or remaining inactive for most of the day with little or no exercise at your job or during your leisure time.

1 Less than 1500 calories

2 1500 to less than 3000 calories

3 3000 to less than 4500 calories

4 4500 calories or more

Perceptions of caloric labeling in chain restaurants

1. Do you feel that you know enough about your daily caloric requirements to make lower calorie choices in chain restaurants or do you feel that you don't know enough?

1 Yes, know enough

2 No, do not know enough

2. If you were told the calorie count of foods at the point of purchase in a chain restaurant, such as McDonalds or Subway, how useful would that information be to your food choice? Would it be...?

1 Very useful

2 Somewhat useful

3 Not very useful

4 Not at all useful

3. Would you be more or less likely to eat at chain restaurants if they reported calorie information on the menus alongside the price for each food item?

1 More likely to eat at chain restaurants reporting caloric information alongside price

2 Less likely to eat at chain restaurants reporting caloric information alongside price

3 Not more or less likely to eat at chain restaurants reporting caloric information alongside price

4. A few cities are now requiring some chain restaurants (such as McDonalds or Subway) to post calorie information on the menu boards next to the price for each food item. If you were in a chain restaurant which had calories posted on the menu board next to the price for each food item, would that encourage you to...

1 Select a higher calorie food

2 Select a lower calorie food, or

3 Would it not influence your food purchase at all?

5. If you were in a chain restaurant which had calories posted on the menu board next to the price for each food item, and you selected a higher calorie food, would you feel guilty for picking a higher calorie food or would you not feel guilty?

1 Yes, would feel guilty for picking a higher calorie food

2 No, would not feel guilty for picking a higher calorie food

6. If you were in a chain restaurant which had calories posted on the menu board next to the price for each food item, would you be more likely to pay attention to the price or to the calories?

1 Pay attention to the price

2 Pay attention to the calories

*Perceptions of calorie posting legislation*

1. Do you favor or oppose the government requiring chain restaurants, such as McDonalds or Subway, to post calorie information on menus or menu boards for each food item at the point of purchase?

1 Favor

2 Oppose

2. Do you think the federal government should be responsible for requiring chain restaurants (such as McDonalds or Subway) to post calories on menus next to the price for each food item or do you think it should be the responsibility of state and local governments?

1 Responsibility of the federal government

2 Responsibility of state and local government

## Pre-publication history

The pre-publication history for this paper can be accessed here:

http://www.biomedcentral.com/1471-2458/10/121/prepub
